# Evidenced Interventions Supporting the Psychological Wellbeing of Disaster Workers: A Rapid Literature Review

**DOI:** 10.3390/ijerph22091454

**Published:** 2025-09-19

**Authors:** Carolyn Deans, Shannon Carter

**Affiliations:** 1 Australian Psychological Society, Melbourne 3000, Australia; 2 Faculty of Education, Monash University, Melbourne 3800, Australia

**Keywords:** psychological well-being, psychological health, mental health, intervention, support, disaster, emergency, crisis, frontline workers, emergency services

## Abstract

This rapid literature review was conducted to better understand the evidence base for interventions aimed at improving psychological well-being in disaster response workers. Three databases were searched: MEDLINE, APA PsycArticles, and Embase. Grey literature reviewed included results of a Google Scholar search and organisation-recommended reports and articles. Of the 959 screened records, 25 studies were included, 13 of which evidenced the benefit of the studied intervention, and two included screening tools to identify at-risk workers. The results showed that evidence-based interventions exist to support disaster response workers to varying degrees in terms of perceived stress, anxiety, depression, post-traumatic stress symptoms, burnout, sleep quality, somatic symptoms, irrational performance beliefs, and emotional and social well-being. Identified interventions featured neurofeedback, psychoeducation, mindfulness, reflective practice, and adjustments to cognitions or behaviours. Interventions varied in delivery (online with pre-recorded content, asynchronous, and guided learning), context (delivered to groups, individuals, and in work or private settings), and facilitator (psychologists, mental health practitioners, and medical doctors). Several interventions improved aspects of psychological well-being in disaster response workers; however, most findings were produced by quasi- or non-experimental designs, suggesting further research is required to clearly ascertain their benefits.

## 1. Introduction

Disaster support workers are exposed to events that have the potential to negatively and sometimes traumatically affect psychological well-being. While supporting disaster-struck communities, workers can be exposed to material that can induce fear, horror, and helplessness, and may encounter work overload, low role clarity, physically demanding work conditions, and difficult interpersonal situations, including distress and abuse. Consequently, disaster response workers have an elevated risk of experiencing post-traumatic stress disorder (PTSD) [1], anxiety and depression [2], burnout [3], compassion fatigue [4], and a range of other psychosocial risks. Those who work in ad hoc disaster relief roles, such as members of the community who volunteer, also have an even greater risk due to their lack of preparation and training for traumatic exposure [5,6]. Evidence also suggests that as potentially traumatic events accumulate, the risk they pose to psychological well-being increases [1]. Psychological well-being is understood not only as the absence of a diagnosable mental health disorder, but also through the individual’s ability to function and sustain performance following potentially traumatic exposure. This is consistent with the World Health Organisation’s understanding of mental health. This evidence is especially important when considered in tandem with the rise in disaster frequency. Due in part to climate change, the volume of environmental disasters has increased steadily in the past decades [7]. In the Intergovernmental Panel on Climate Change’s most recent report, they confirmed that the magnitude of natural disasters is increasing and currently exceeding past projections. These shifts also threaten the emergence of additional infectious diseases [8]. It is predicted that disaster response workers will both experience an increased need for their services and potentially increased risk to their psychological wellbeing. There is a consequent need to understand which interventions can enhance or mitigate risks to their psychological well-being and maintain a healthy workforce in the face of disasters.

Disaster response workers include a range of roles incorporating staff or volunteers who operate in the context of disasters, emergencies, or community events in frontline, community, or support roles [9]. The literature on appropriate interventions to mitigate mental health risks in these workers is still emergent [10]. Psychological responses that support full-time employed first responders, such as police officers, are emergent [11,12], and although there are evidence-based interventions for those who have developed a mental health disorder, a 2021 systematic review of interventions for first responders concluded early intervention or preventive approaches have not been proven [13]. In addition, the question of which responses are suitable for the broader cross-section of disaster response workers is not defined [14].

A recent meta-analysis attempted to explore interventions for trauma-exposed workers via a meta-analysis of efficacy trials (quasi-experimental or randomised) of existing and new interventions published from the year 2008 to the year 2019 [15]. The inclusion criteria mandated that the studied population be any adult workers (not just frontline or emergency support) exposed to potentially psychologically traumatic events. The 42 studies included suggested that interventions can reduce anxiety, problematic heart rate, maladaptive coping, stress, and psychological symptom burden. However, the included studies were often not set in the context of disasters. As the inclusion years end in 2019, it did not include studies produced because of the COVID-19 pandemic, which was declared in December 2019. Given the prevalence of psychological harm incurred by healthcare professionals when they are involved in natural disaster events and the increase in exposure to fear, horror, and helplessness in the context of overwork during the pandemic [16], it is reasonable to anticipate that interest in this area of research would increase. A more recent systematic review focused on psychological interventions aimed to support healthcare workers (not necessarily frontline) during COVID-19 [17]. Perhaps due to the social distancing imperative of a contagious pandemic, most interventions found were delivered digitally or by mail. However, the two that yielded the best results were conducted with frequent contact, feedback for the client, and according to the authors, resembled psychological resources more than interventions, as they were only effective with additional psychotherapy or psychotropics. Consequently, the overall findings of the review were limited, but promising for those seeking helpful resources to disseminate in conjunction with other traditional interventions.

A gap in the literature is a review of the evidence in support of interventions targeting the psychological well-being of those who support their community in the aftermath of disasters and emergencies, including health emergencies. In this review, these people are defined as disaster response workers. The review aimed not only to understand which interventions yield positive results, but also how these interventions are implemented and evaluated. A formal enumeration of the research questions is as follows:

1. What interventions are used to enhance psychological well-being among disaster response workers?
2. How are these interventions implemented and evaluated?
3. What is the evidence of the effectiveness of these interventions?

## 2. Materials and Methods

### 2.1. Design

A rapid literature review design was used to achieve the primary aim of exploring the best-practice or evidence-informed methods or interventions for enhancing psychological well-being among disaster response workers. This review methodology is a modification of a systematic literature review, which allows a degree of efficiency by restricting the scope of content, time, or other factors [18]. This brevity was a desirable trait for the present study, given budget and time limitations. It was achieved by limiting the scope of inclusion to non-military contexts and the timeframe of articles to the past decade (2014–2024). The selection of databases was also limited to reduce the amount of overlap of article selection and the number of non-health-related topics, by selecting databases with focused coverage of biomedicine and psychology. No other changes to the systematic review process were made. This rapid review was conducted in accordance with the PRISMA (Preferred Reporting Items for Systematic reviews and Meta-Analyses)reporting guidelines.

#### Risk of Bias

While a formal risk of bias assessment was not within the scope of this rapid review, a simplified evaluation was made. This evaluation was based on: selection and randomisation of participants; use of a control group; outcome measurement (use of validated tools); follow-up timeframe; and statistical analysis. For the Delphi study, the evaluation was based on: selection process, management of confounds, analysis, and outcome reporting. Given the lack of measurement, descriptions of programs were categorised as high risk. Studies on measures were evaluated based on: sample representativeness and size; translation rigour (where applicable); use of comparison scales; statistical analysis; and reporting transparency. Effect sizes are as reported by the authors. Almost all studies used Cohen’s [19] guidance on η^2^ scores to assess small, moderate, or large effects; one study used Cohen’s benchmarks for d, adapted for η^2^, and one study used odds ratios for logistic regression, based on Cohen’s guidance.

### 2.2. Inclusion and Exclusion Criteria

The necessary features for inclusion in the review were: English-language peer-reviewed psychology journal articles published between 2014 and 2024 (inclusive) that evidenced interventions supporting the psychological well-being of disaster response workers.

Articles that were not disaster-related, studied groups under the age of 18, or featured interventions that addressed the psychological well-being of the general public rather than workers, were excluded. In addition, articles that contained the term ‘military’ or content about the military were also excluded, as this population is likely to differ from civilian workers in both the types of trauma they are exposed to and the organisational context.

To explore a breadth of interventions, eligible psychological well-being outcomes were defined as aspects of: mental health, coping under stress, and absence of mental illness.

### 2.3. Information Sources

#### 2.3.1. Published Literature

Information sources for the published literature were MEDLINE, APA PsycArticles, and Embase. Each database was searched via the Ovid interface using the following search formula:

(((‘Psychological wellbeing’ or ‘Psychological health’ or ‘Mental health’) and (‘intervention*’ or ‘support*’ or ‘promotion’ or ‘informal check*’ or ‘wellbeing-check*’ or ‘well-being-check*’ or ‘wellbeing check*’ or ‘well-being check*’ or ‘wellbeing support’ or ‘well-being support’ or ‘informal check*’ or ‘behavioural activation’ or ‘training’ or ‘informal check*’ or ‘professional guidance’ or ‘psychological first aid’ or ‘psychological first-aid’ or ‘screening’ or ‘psychoeducation’ or ‘psychological education’ or ‘psychosocial’) and (‘disaster*’ or ‘emergency’ or ‘crisis’) and (‘worker*’ or ‘volunteer*’ or ‘frontline’)) not ‘military’).af.

**Figure 1:**
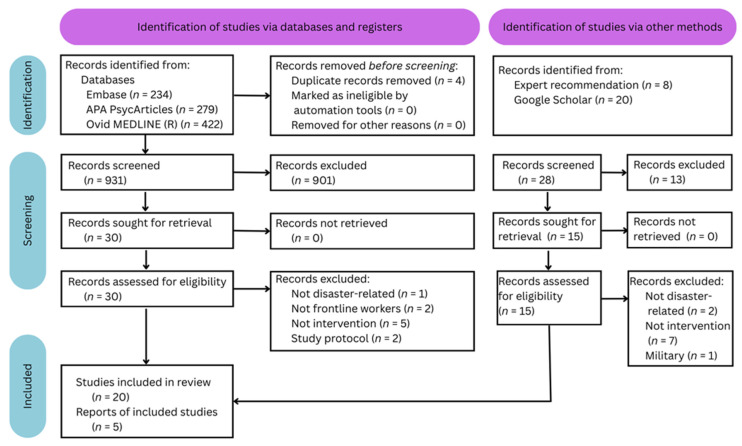
Prisma Identification, Screening, and Inclusion Flow Chart.

All returns were exported to CSV files, merged, and screened for duplicates using Microsoft Excel’s ‘fuzzy lookup’ plugin. The screening process included a sense-check of all returned article titles to eliminate duplicates not identified by Microsoft Excel and those that did not meet the inclusion criteria. In cases where the title was ambiguous, the abstract was read instead. This round of screening was conducted by one author. The second author reviewed the title and abstract of 10% of all records (included or excluded), randomly selected. The results of these independent checks were compared to identify human error or rater bias; the spot check did not reveal error or bias. Disagreements between decisions were resolved via discussion after each author read the full article. The first author had the final decision where required. A small number of articles that seemed to meet the inclusion criteria during these checks were also excluded while reading the full article (see Figure 1).

#### 2.3.2. Grey Literature

A Google Scholar search was conducted to ensure publicly available, relevant grey literature was included. As this search returned an excessive number of results and there are no standardised guidelines on cutoff points, the authors decided to review the first two pages of search results (100 links). An expert on occupational mental health within the commissioning organisation identified additional references commonly used in the Australian industry to guide interventions. Both these sources were assessed using the inclusion and exclusion criteria for the published literature (minus the peer-reviewed article criteria) before being added.

## 3. Results

Data extraction and selection (see Figure 1) produced a total of 25 studies (see Table 1), 13 of which provided evidence for using an intervention to support the psychological well-being of disaster response workers.

### 3.1. Interventions Supporting Workers Without Manifest Symptoms

#### 3.1.1. Critical Incident Stress Debriefing

An observational study explored a Critical Incident Stress Debriefing (CISD) intervention delivered to German emergency response workers who responded to a terrorist attack [20]. CISD is a debriefing session delivered to small groups 24−72 h after the incident, which includes in sensu exposure to each group member’s concerns. Of the overall sample (*n* = 55), 37 participated in CISD. Four months after the event, stress and quality of life were assessed via the German versions of the Patient Health Questionnaire (PHQ-D), the WHO Quality of Life Questionnaire (WHOQOL-BREF), the Posttraumatic Stress Disorder checklist (PCL-5), and the Brief Symptom Inventory (BSI). Those who received CISD exhibited lower psychological health and higher depressive symptomatology after the event, with some showing higher phobic anxiety after the event. These metrics were not measured before the intervention; therefore, there is no statistical comparison and limited conclusions can be drawn.

#### 3.1.2. Rational Emotive Behavioural Coaching

One study explored the effects of Rational Emotive Behavioural Coaching (REBC) on 34 UK-based frontline fire workers (18 firefighters, six crew members, seven watch managers, two station managers, and one group manager) [21]. REBC is a psychological intervention that aims to ameliorate irrational cognitions via assisted problem solving and cognitive restructuring, in this study, in relation to work circumstances. The study used an experimental pre-post design where the treatment group received one-on-one REBC sessions weekly and bi-weekly for twelve weeks, while the control group received no intervention. The dependent variables of interest included irrational performance beliefs, resilience, chronic stress, depression and anxiety, and ‘presenteeism’. REBC reduced irrational performance beliefs in the sample and this reduction was maintained at the 3-month follow-up with a large effect size. No other dependent variables were found to have been influenced by the intervention.

### 3.2. Interventions Supporting Workers with Sub-Clinical Symptoms

#### 3.2.1. Intensive Neurofeedback Protocol

One pilot study tested the efficacy of 10 × 20-min neurofeedback sessions delivered over two weeks to increase alpha wave values in medical doctors on duty during the COVID-19 pandemic [22]. Neurofeedback utilises real-time electroencephalographic feedback to assist participants in reinforcing ‘desirable’ brain states. This study hypothesised that increasing alpha wave values would improve sleep and reduce stress-related symptoms. While the study did not find a reduction in stress-related symptoms, self-report responses indicated that it improved sleep quality in the sample of 18 doctors, with a large effect size.

#### 3.2.2. Stepped-Care Models (Variants of Psychological First Aid and Cognitive Behavioural Therapy)

One analyst-blind, parallel, multicentre RCT evidenced the benefits of a stepped-care program aiming to reduce anxiety, depression, and post-traumatic stress symptoms among Spanish healthcare workers at 7, 13, and 21 weeks after baseline [22]. It was delivered remotely via a mobile website and up to 15 min of telephone or message contact per week. Stepped-care interventions aim to progressively allocate tiers of non-professional, professional, and specialised treatment based upon the manifest needs of the individual. The intervention included enhanced care as usual in the form of psychological first aid; a self-help style mobile intervention for the treatment group, which included mindfulness and cognitive therapy techniques; and Problem Management Plus, a cognitive-behavioural intervention for workers with significant psychological distress. The content of Problem Management Plus includes problem-solving techniques, social support enhancement strategies, and cognitive techniques. The stepped-care treatment was more effective at reducing anxiety and depression symptoms compared to the *enhanced care as usual* group, but roughly equivalent at reducing post-traumatic stress symptoms. Effect sizes were low to moderate for those who completed the self-help intervention, and moderate to large for those who completed Problem Management Plus.

### 3.3. Interventions Supporting Workers with Clinical Symptoms

#### 3.3.1. Urgent Eye Movement Desensitisation and Reprocessing (URG-EMDR)

One pilot study [24] delivered URG-EMDR via the ‘butterfly hug’ method (tapping alternate shoulders with crossed arms) to nurses (*n* = 17) experiencing professional stress related to their COVID-19 workload and who showed clinical signs of anxiety or depression. EMDR combines cognitive therapy and traumatic memory exposure techniques with bilateral stimulation to reduce memory intensity and impact. All participants had previously received EMDR therapy and therefore already knew some of the techniques. URG-EMDR was delivered in a single session (average time 2.14 h) over video call. The intervention showed significant reductions at 24 h and 1 week follow-up in anxiety, depression, subjective units of distress, fear of going to work, and fear for safety at work. Improvements in fear for safety at work and subjective units of disturbance started to diminish by the 1-week follow-up, while the other outcomes continued to improve. However, no effect sizes were reported, and the sample was very small.

#### 3.3.2. Culturally-Relevant Jungian Archetypes

One article explored the impact of an intervention based on Jungian psychology that used culturally relevant symbolism to increase the resilience of 14 Physicians and 29 Nursing Staff in China during COVID-19 [25]. In particular, the gourd, which is heavily referenced in historical Chinese culture, was used for its imagery around sealing, housing, protection, healing, and transformation. The intervention, delivered optionally within an existing psychotherapy context, involved asking the client to imaginatively engage with the proposed gourd-archetype imagery and lasted roughly 15 min. The study used a repeated cross-sectional design with some case study data. There were several improvements across various mental health variables (e.g., anxiety, depression, sadness). As the study was not experimental, it did not report effect sizes and did not control for the benefits provided by the existing psychotherapy, historical changes, or other variables.

### 3.4. Education and Preparation Models

#### 3.4.1. Mental Health Literacy and Stress Management Psychoeducation

Two interventions tested the efficacy of psychoeducation on mental health literacy and cognitive-behavioural stress management. One intervention combined psychoeducation and self-help resources with group counselling and a psychological care hotline for front-line nurses working in isolation wards during COVID-19 [26]. Nurses were provided the education one to three days before entering the isolation wards, including two 1-h face-to-face sessions on mental health literacy, relaxation skills, and sleep hygiene. Self-help videos and audio with further psychoeducation on insomnia, stress, and emotional disturbance were supplied. While they were in 14-day quarantine, nurses received four 2-h online group supportive counselling sessions and continued access to a psychological assistance hotline. At the end of quarantine, somatic symptoms and anxiety were lower in participants compared to the day before entering and the day after leaving the wards. Depression symptoms were lower compared to the day after leaving the wards. There was no pre-intervention measurement of the sample’s primary outcomes, no control group, and no effect sizes reported. Another intervention [27] tested the efficacy of a series of four psychoeducational videos in reducing anxiety and depression among healthcare professionals (*n* = 440) working in state hospitals during COVID-19. The video content was diverse and covered the following subjects: anxiety, depression, psychological resilience, substance use, being aware of and controlling psychological reactions, problem-solving skills, mindfulness strategies, and relaxation techniques. Self-reported depressive symptoms decreased significantly in those who use alcohol and those who had less than four work shifts per month, but anxiety symptoms did not change, and no effect sizes were reported. Measurements were only taken before any videos were watched and after all were watched, thus it is unclear which videos or content had an impact.

#### 3.4.2. Cognitive Behavioural, Mindfulness, and Acceptance Training

Four studies explored training in cognitive, behavioural, mindfulness, or acceptance techniques before exposure to difficult events. The efficacy of the Resilience@Work mindfulness program was explored via a cluster randomised controlled trial (RCT) with 143 firefighters stationed across 24 fire stations in Australia [28]. The intervention was based on mindfulness, acceptance, and self-compassion. Acceptance techniques aim to encourage psychological flexibility in recipients by training them to non-judgmentally accept the presence of emotions. The intervention was delivered online (tablets were provided at the fire stations) and consisted of six training sessions of 20–25 min. The content employed video, audio, and interactive exercises to teach skills such as how to manage uncomfortable thoughts via cognitive defusion. Mindfulness tracks were made available for mobile download to encourage continued practice. The control group was given access to six sessions with guidance on health and well-being topics. Primary outcomes included: bounce-back resilience measured via the Brief Resilience Scale, and adaptive resilience measured via the Connor–Davidson Resilience Scale. Secondary outcomes were mindfulness, cognitive fusion, experiential avoidance, psychological inflexibility, self-compassion, optimism, coping styles, and sense of purpose. The treatment group had improvements in adaptive resilience scores, double that of the control, with a moderate-to-large effect size. Participants with the most improvement in adaptive resilience were those who underwent the most mindfulness program sessions. Several secondary outcomes were also improved by the intervention at the 6-week follow-up, including optimism, use of emotional support, and use of instrumental support; however, only mindfulness skills persisted until the 6-month follow-up. A similar mindfulness education intervention developed in Madrid, Spain, was intended to improve emotional regulation in front-line healthcare workers [29]. Each intervention session lasted 5–10 min, delivered on-site in COVID-19 wards, and made available to participants twice a day, every day of the calendar week, over 7 weeks. Participants were trained in breathing, soft hatha yoga stretches, sentences, and gestures that engage in compassion towards self and others, and to identify and non-judgmentally accept their emotions, thoughts, and bodily sensations. Participants were asked to rate how well the intervention reduced their current stress and their mean response was 8.4 out of 10, but there was no statistical difference in perceived helpfulness between one session and more than one session, and no other statistical analysis was reported. Three participants reported minor adverse effects such as dizziness or increased anxiety. The questionnaire sample comprised only 150 participants of the 1,000 participants of the intervention.

The web-based My Health Too intervention [30] provided psychoeducation and training in mindfulness, acceptance, behavioural and coping strategies, action towards values, barriers to the use of self-compassion, soothing difficult emotions, and sleep management. The intervention consisted of seven 20-min video sessions. At the end of each video session, participants (*n* = 70) were offered a call with a psychologist and encouraged to practice skills via homework materials. The control group (*n* = 77) received bibliotherapy. Compared to baseline, perceived stress improved significantly at all follow-ups (8, 12, and 24 weeks). However, of the nine outcome variables, work-related rumination was the only one to see a significant improvement compared to the control, and no effect sizes were reported. Similarly, an RCT (with waitlist control) tested the efficacy of the Disaster Worker Resiliency Training (DWRT) program [31]. DWRT is a 4-h workshop on definitions of resilience, acute stress symptoms, and how to identify when it is time to engage in help-seeking; common stress reactions; recovery trajectories and safety, health, and risk factors related to the relevant disaster; and nutrition, exercise, recuperative sleep, social support, health behaviour goals, and relaxation information. The intervention also aimed to encourage help-seeking via employer and community resources. It was tailored to first responders’ unique concerns, such as privacy and cultural values relating to protecting others. At 3-month follow-up, DWRT participants showed improved healthy lifestyle behaviours, stress management, and spiritual growth compared to the waitlist control, with moderate effect sizes. Those in the treatment group were less likely to report increases in perceived stress in response to reported trauma exposure, with a moderate effect size.

#### 3.4.3. Psychological First Aid Training

Psychological First Aid (PFA) training teaches skills in psychosocial support to promote natural recovery after an emergency, disaster, or traumatic event. It involves helping people feel safe, connected to others, calm, hopeful, and self-efficacious, and ensuring access to physical, emotional, and social support. An observational, pre-post pilot evaluation design explored the effects of PFA training on 56 adult COVID-19 frontline workers serving American Indian and Alaska Native (AI/AN) communities [32]. Of these, 75% were AI/AN themselves. A collaborative work group adapted the intervention to the AI/AN community, including culturally sensitive language updates and the inclusion of cultural practices, values, and teachings. The culturally adapted version consisted of an online resource guide and four training modules. There were significant increases in positive mental health and social well-being, and decreases in burnout for participants at the 3-month follow-up, but no effect sizes were reported for these changes. There were no significant changes in communal mastery, coping skills, anxiety, perceived stress, emotional well-being, or psychological well-being at follow-up. A second study utilised a parallel-group, assessor-blinded, cluster-RCT (*n* =1399) to assess the effects of one-day system-based PFA training on a sample of 690 frontline medical workers from 42 hospitals in China [33]. The intervention was an amalgamation of the Johns Hopkins University and the World Health Organisation PFA guidelines, adapted to the Chinese cultural context with local examples, role plays, case studies, cultural beliefs, and values. At the 2-month follow-up, the study found improvement with a moderate effect size in PFA skills, knowledge, and attitude. Small improvements were found in the secondary outcome general self-efficacy (low effect size); no other significant changes in secondary outcomes (post-traumatic growth, professional quality of life) were found.

### 3.5. Reflective Practice (Supervision and Peer Support)

#### 3.5.1. Integrated Supervision Model

A Delphi (expert consensus) study aimed to fill research-to-practice gaps about supervision quality and purpose in the context of mental health support to stakeholders working in response to humanitarian crises [34]. The majority (65%) of the international sample (*n* = 48) had experienced both being supervised and giving supervision. Participants were recruited to provide their agreement or disagreement with 28 statements related to supervision practices in emergency contexts. Consensus (75% or above) was reached on 21 of 28 statements regarding supervision. Items with the most consensus stated that active listening is an important feature of skilful supervision, multiple tiers of supervision lead to optimal functionality, supervision in the context of mental health support should focus on coaching or teaching specific skills, and interpreters who engage in supervision sessions should also have access to supervision.

#### 3.5.2. Peer Support

The CopeColumbia intervention aims to provide peer psychological support to promote psychological well-being and prevent burnout, acute distress disorder, and depression [35]. Three delivery methods were included in the study: peer support groups facilitated by psychologists and psychiatrists lasting 30 min; one-to-one peer support programs lasting 20 min; and staff education sessions lasting 30–60 min to accommodate large groups. Groups met between one and five times. In these sessions, attendees discussed the difficulties they faced in their work, and the facilitators identified themes that could be addressed by evidence-backed techniques such as labelling and validating emotions. While qualitative feedback suggests the program was helpful, severe issues with study design (e.g., those relating to historical validity and uncleaned data) render it difficult to evaluate.

### 3.6. Organisational Frameworks

#### 3.6.1. Trauma-Informed Practice

While not assessing efficacy, one journal article reported on the application of an existing intervention, the Witness-to-Witness Program (W2W), adapted for both English and Spanish speakers in a COVID-19 context [36]. W2W is underpinned by the witnessing model, a theory that posits psychological distress is produced by ineffectual action caused by epistemic or resource-related shortcomings. The purpose of W2W is to elevate people to an ‘aware and empowered’ position via psychoeducational webinars, clinician listening sessions, open-enrolment peer support groups, and organisational consultations to foster trauma sensitivity in the workplace.

#### 3.6.2. Moral Injury Prevention

One expository paper puts forth suggestions to reduce the impact of moral injury on the psychological well-being of healthcare workers during COVID-19 [37]. Moral injury is defined as a form of psychological distress produced by witnessing actions that violate the individual’s moral or ethical code. The paper made suggestions about how to reduce moral injury in professions at risk of experiencing this phenomenon. It recommended training on how to approach likely moral dilemmas, reduce false assurances, and provide psychoeducation about moral injury, burnout, and their consequences. It also recommended ongoing supervisory support; routine support services which feature reporting of moral injury; and vigilance on the part of leaders towards the avoidance of communicating feelings of shame or guilt. It contained no experimental or real-world program.

#### 3.6.3. Preparation, Response, and Recovery

A 2020 literature review of existing studies aimed at enhancing the psychological well-being of disaster responders who had not received formal training to respond to a natural disaster qualitatively synthesised the findings into a series of recommended actions [38]. This article had a particular focus on grey literature produced in Japan. Actions included those to be taken by workers and organisations before, during, and after a disaster. Actions were aimed at three outcomes: rendering stressors manageable by helping workers understand them; alleviating chronically stressful circumstances; and responding to crises. An example recommendation meeting the first goal is to “assess the readiness of one’s health, work, and family for enrolment”. An example of the second goal is to “develop a peer support system within the team”. An example of the third goal is “take rest until recovery from the mental health crisis”.

#### 3.6.4. Whole-of-Organisation

The Beyond Blue Good Practice Framework for Police and Emergency Services Organisations [12] was informed by qualitative and quantitative evidence, most often qualitative. The document describing the framework is grey literature and not itself a study. Recommendations made by the framework structure a list of five higher-order actions, with each accompanied by a list of lower-order actions. The first higher-order action is to systematically manage risk. For example, it is recommended that organisations develop processes to monitor exposure to trauma and screen for PTSD symptoms. The next action is to prepare and roll out a strategy for promoting mental health and well-being in the workplace, for example, by encouraging a self-reflective culture in the organisation. Thirdly, organisations are encouraged to increase leadership engagement and competency. This takes many forms, such as training leaders to identify antisocial conduct, promote values such as honesty and trust, and foster a sense of belonging amongst their team. Fourthly, organisations are called on to reduce stigma, for example, through leaders speaking of mental health candidly in the workplace. Finally, it is recommended that organisations implement mental health and well-being training that is evidence-based, avoids repeated content, and remains relevant to the workers’ roles.

The Australian Red Cross produced a brief overview of their approach to protecting the well-being of their frontline and emergency support workers during emergencies [39]. This approach follows the stepped care approach of the Beyond Blue Good Practice Framework and includes screening for at-risk workers, encouraging a culture of transparency around matters of well-being, and creating roles within the organisation that support the maintenance of well-being in the workforce. Pre-deployment written material provides a realistic preview of what to expect in the disaster context and the conditions the worker may face.

### 3.7. Measures Identifying Vulnerable Workers

Although measurement tools are not interventions, they have the potential to point to new areas for intervention. They were therefore included in this review and separated into a discrete category.

#### 3.7.1. Symptoms—Compassion Fatigue and Self-Triage for PTSD

One study tested the psychometric properties of a Mandarin-adapted version of the Compassion-Fatigue-Short Scale (CFSS) in medical workers and firefighters in Shanghai [40]. Compassion fatigue is a state of exhaustion, possibly brought about by traumatic stress, that reduces the individual’s ability to experience empathy and compassion towards others. The new scale consisted of two subscales, secondary trauma (5 items) and job burnout (8 items). The scale demonstrated construct and predictive validity as well as internal consistency. Exploratory factor analyses showed that job burnout and secondary trauma were associated with compassion fatigue in these emergency workers.

The ability of the Psychological Simple Triage and Rapid Treatment (PsySTART) scale aims to assist self-triage for PTSD and depression based on 14 risk factors. Its validly was tested with frontline workers after Typhoon Haiyan in the Philippines [41]. Results showed that the number of potentially traumatic events a worker is exposed to predicted PHQ-8 and PCL-5 scores in the sample approximately four months later. Results also suggested that low self-efficacy relating to assigned tasks and perceived (or actual) risk of harm to oneself or coworkers predicts the occurrence of PTSD symptoms.

#### 3.7.2. Resilience Processes—Coping Style and Perceived Competence

The relationship between healthcare providers’ frequency of coping styles in the context of COVID-19 was explored using the Brief COPE Inventory [42], a 28-item survey that asks respondents to indicate on a Likert scale their frequency of use of specific coping styles. The timeframes included in the survey were adjusted to capture the COVID-19 pandemic. The coping strategies included in the survey were grouped into tiers of ‘problem-focused’ (e.g., problem solving or cognitive restructuring) or ‘emotion-focused’ (e.g., emotional expression or avoidance) styles. Secondary variables were captured via: the Work and Well-Being survey, Stress Appraisal Measure, a Generalised Anxiety Disorder scale, and the Screening Tool for Psychological Distress. Both higher levels of threat appraisal and emotion-focused coping were significant moderate predictors of depression and anxiety in response to their frontline work. These variables predicted anxiety and depression, while other expected predictors (e.g., availability of personal protective equipment and hands-on patient care) did not. An anxiety response was also found to predict depression. Problem-focused coping styles were also weakly positively correlated with anxiety and engagement, and enthusiasm towards work.

A Korean study developed and validated the Perceived Competence Scale for Disaster Mental Health Workforce (PCS-DMHW) [43] via focus group interviews (N = 48; nine psychologists, nine social workers, six psychiatrists, 11 nurses, two public officials, and 11 volunteers). Participants discussed their experiences in disaster site management, case management, and the human resource and psychological support they received. Education, training, and competency were discussed at individual and organisational levels. Findings led to the first formulation of (English-language) items in the PCS-DMHW. The final PCS-DMHW was comprised of an individual competence subscale (15 knowledge and skill-related items; nine attitude-related items), an organisational competence subscale (12 teamwork items; six network items; three ‘followship’ items), and a supplementary subscale (three items) related to burnout prevention. The final version of PCS-DMHW was redistributed to 40 workers for completion. The PCS-DMHW presented good internal consistency, construct, and convergent validity. Test-retest reliability, however, was slightly below acceptable thresholds.

### 3.8. Quality of Studies and Effect Sizes

Among the studies reviewed, 13 reported improvements in at least one psychological well-being outcome, while two successfully screened for risk factors associated with poor outcomes following disaster exposure. However, only the RCT studies and the study translating an already-validated measure were assessed as being high quality (low risk of bias). Most studies in this review had low-quality methodologies with a moderate risk of bias.

Effect sizes were only reported for seven studies in this review. The two large effect sizes reported were for REBC on irrational performance beliefs and neurofeedback on self-reported sleep quality. The four treatments with the most improvement reported saw benefits of 9−42% of the scale’s upper limit at the final follow-up on their respective outcome variables, but three of these were quasi-experimental studies. These outcomes were a reduction in irrational performance beliefs (REBC), a reduction in anxiety among those aged over 55 (psychoeducational videos), and a decrease in depression and anxiety symptoms (URG-EMDR). The only RCT with significant improvement in scale scores was psychoeducation with Problem Management Plus, which saw a reduction in anxiety, depression, and PTSD symptoms, and had a low-moderate effect size for the psychoeducation and a moderate-large effect size when Problem Management Plus was added. The online mindfulness education program saw moderate to large effect sizes for the difference between improvements for the control and treatment groups.

## 4. Discussion

There is growing interest in interventions aimed at supporting the psychological well-being of disaster response workers. This rapid literature review of the past decade of work highlights a focus on psychoeducation, cognitive-behavioural training, mindfulness, and neurofeedback interventions. Despite this expansion, robust experimental evidence for their effectiveness remains relatively scarce.

### 4.1. Evidence-Based Interventions

These interventions targeted a wide range of outcomes, including perceived stress, anxiety, depression, post-traumatic stress symptoms, burnout, sleep quality, somatic complaints, irrational performance beliefs, and emotional and social well-being.

The RCT interventions studied: Stepped care with a low-level CBT intervention; online mindfulness training; online CBT psychoeducation; and a CBT-based psychoeducation program. These all showed an ability to reduce symptoms associated with work stress, such as healthy lifestyle behaviours, perceived stress responses, anxiety symptoms, and work-related rumination. However, none showed a reduction in mental health disorders or post-traumatic stress symptoms, and one reported a decrease in effect over a longer follow-up. There are small evidence-based benefits of online PFA training (culturally adapted or manualised) in improving PFA knowledge, but mixed effects on its ability to address well-being factors.

Quasi-experimental studies point to only small effects for a range of interventions. Online mental health literacy, psychoeducation, and supportive counselling programs may yield small improvements in anxiety, depression, and somatic symptoms; however, one study on psychoeducation found an increase in depression for those with only a high school education. The benefits of mindfulness, EMDR, and neurofeedback have initial qualitative or pilot evidence. CISD continued to reflect past research, with problematic observational findings. Neither rational emotive behavioural skills nor Jungian archetypes showed effectiveness for any well-being factor.

### 4.2. Group, Leader, or Organisational Approaches

A disaster preparation education intervention and two screening tools (for compassion fatigue and potentially traumatic events) enhanced leaders’ capacity to support disaster workers. It does not appear, however, that there has been any innovation in organisational frameworks or stepped care models that change the recommendations for organisations. There were no interventions focused on group approaches such as peer support, which is surprising given the evidence suggesting social support is a good predictor of psychological health in police, firefighting, and medical professions [44].

### 4.3. The Impact of COVID-19

Interventions most often focused on healthcare workers and the COVID-19 pandemic contexts. It is clear that the COVID-19 pandemic had a significant impact on the well-being of healthcare personnel [45]. The context of this disaster and the workforce involved are different from other natural and human-related disasters, and generalising about findings should occur conservatively. However, controlled trials of mindfulness and cognitive skills showed similar efficacy in reducing stress responses for healthcare workers, firefighters, and disaster responders. One potential effect of the COVID-19 crisis is that, perhaps due to the safety concerns related to transmission of the virus, many interventions explored were delivered remotely with little impact on effectiveness. Some took an asynchronous delivery approach and one such intervention was the most effective of those examined at reducing anxiety.

### 4.4. Strengths and Limitations

Due to the nature of a rapid review, there were limitations related to the exclusion criteria. For example, non-English language papers and those published over 10 years ago were excluded. While this timeframe was selected for pragmatic reasons, it is acknowledged that excluding pre-2014 literature may omit relevant earlier findings, potentially introducing bias. This is mitigated by the fact that any identified literature reviews included in the current study were read to provide relevant context, and any significantly efficacious intervention would be more likely to have been replicated in the past decade.

While the scope of the search was limited to three databases (MEDLINE, APA PsycArticles, and Embase), these were chosen for their relevance to the topic. Broader databases were omitted due to resource constraints, which may exclude relevant studies from other disciplines. A Google Scholar search and recommended grey literature were added to the search, and this can be considered a strength compared to other rapid or systematic literature reviews. The lack of geographic restriction in the exclusion criteria is also atypical for rapid literature reviews.

As the present study included interventions supporting workers facing all forms of disaster and all psychological well-being variables, the breadth of studies reviewed is broader than other recent systematic reviews on related topics [13,46,47,48,49,50,51]. The study also included interventions tested with any design (rather than just quasi-experimental designs), making it more inclusive than the relevant predecessor. A consequence of this, however, is that several of the included studies possessed weak designs that restrict the confidence with which conclusions can be drawn. Finally, this review added to the literature by examining many recent interventions that did not feature in the most recent review with similar objectives [15].

## 5. Conclusions

It is clear from this review that cognitive and behavioural interventions, with or without mindfulness additions, are the most studied and the most consistent in being able to address aspects of well-being in disaster response workers. Stepped-care approaches using multiple interventions appear to build on this impact and may also have potential as protective measures. An interesting finding, as psychological first aid continues to dominate as a response approach for psychosocial support to the community, is that training in this procedure may have protective benefits for workers as well. This may be due to the link between good role preparation and psychological resilience to traumatic exposure [52]. Related to this, while there were multiple aspects of psychological well-being used as dependent variables, it may be possible from the current review to theorise an assessment battery that predicts poor well-being in response to traumatic events. This would include one with both pre-exposure variables, such as perceived workplace competence, as well as symptom measurement.

This study has found an increase in the past decade in evidence-based interventions supporting the well-being of disaster response workers. Those that have been tested under controlled trials include psychoeducation, cognitive, behavioural, mindfulness, and acceptance approaches, or a combination of these. While the efficacy of three interventions was greater in terms of effect size, each possessed its own design flaw, such as the absence of a control group or the presence of selection bias and, in one case, a risk of harm to less educated populations. Consequently, it is too early for full-throated endorsement of these interventions, and further research is recommended to explore their benefits with a robust and participant-safe study design.

## Figures and Tables

**Table 1:** Summary of Published Literature, Types, and Outcome Variables.

Source	Population	Intervention	Comparison [*Risk of Bias*]	Outcomes
Support to workers without manifest symptoms				
Wesemann et al., 2019 [20]	Firefighters, police, emergency medical technicians + NGOs following a terrorist attack (*n* = 55; Germany)	Critical Incident Stress Debriefing (CISD)	Non-randomised observational study using independent-samples *t*-tests (intervention vs no intervention) [*high* risk]	Psychosocial stressors; quality of life over the past two weeks (physical health, psychological health, social relationships, and environment); post-traumatic stress symptoms; symptoms of psychiatric disorders
Wood et al., 2021 [21]	County Fire and Rescue Volunteers Responsible for Disaster Response (*n* = 34; UK)	Rational Emotive Behavioural Coaching (REBC)	Quasi-experimental (purposeful allocation) pre-post control design [*moderate* risk]	Irrational performance beliefs; resilience; hair cortisol concentration; depression; anxiety; stress; presenteeism
Support to workers with sub-clinical symptoms				
Benatti et al., 2023 [22]	Medical doctors (*n* = 18; Italy)	Intensive Alpha-Increase Neurofeedback Protocol	Pilot efficacy study [*high* risk]	Sleep quality; stress; burnout
Mediavilla et al., 2023 [23]	Health Care Workers with distress (*n* = 115; Spain)	Doing what Matters (stepped care) with Problem Management+ when distress identified	Analyst-blind, parallel, multicentre RCT (TAU, enhanced with PFA) [*low* risk]	Anxiety; depression; PTSD
Support to workers with clinical symptoms				
Tarquinio et al., 2020 [24]	Nurses (*n* = 17; France)	Urgent Eye Movement Desensitisation and Reprocessing (URG-EMDR) single session video call	Quasi-experimental pilot (no control) pre-post efficacy study [*moderate* risk]	Depression; anxiety; subjective units of disturbance; fear of going to work; fear for your safety at work
Liang et al., 2023 [25]	Physicians and Nursing staff (*n* = 43; China)	Gourd Symbolism with Jungian Archetype	Repeated cross-sectional design with some reference to case study data [*high* risk]	Psychologist observed: anxiety; depression; fidgeting; sadness; uncontrollable concern; sense of powerlessness, frustration; tense; insomnia; physical discomfort; guilt and self-reproaching
Education and Preparation				
He et al., 2022 [26]	Nurses working in COVID-19 isolation wards (*n* = 62; China)	Stress Management Training and Group Post-Event Counselling	Quasi-experimental (no control) pre-post study [*high* risk]	Anxiety; depression; somatic symptoms
Yöyen et al., 2022 [27]	Healthcare professionals (*n* = 440; Turkey)	Mental health literacy, cognitive and mindfulness video education	Quasi-experimental (no control) pre-post efficacy study [*high* risk]	Anxiety; depression; challenge; dedication; control
Joyce et al., 2019 [28]	Full-time firefighters from Primary Fire and Rescue and Hazmat stations (*n* = 143; Australia)	Resilience@Work: online-delivered mindfulness education intervention	Cluster pre-post (6-month follow-up) RCT [*low* risk]	Resilience resources; optimism; active coping; use of emotional and instrumental support
Rodriguez-Vega et al., 2020 [29]	Healthcare workers (*n* = 150; Spain)	Mindfulness-Based Crisis Intervention	Exploratory study with post-intervention assessment [*high* risk]	Utility perception; safety and feasibility indicators
Mengin et al., 2024 [30]	Healthcare workers (*n* = 155; France)	My Health Too: online CBT and mindfulness psychoeducation for work stress	Multicentric RCT (bibliotherapy control) evaluation [*low* risk]	Perceived stress; depression; post-traumatic stress symptoms; resilience; insomnia; work-related rumination; credibility of the treatment; satisfaction with treatment; perceived efficacy and utility of sessions
Mahaffey et al., 2020 [31]	Disaster responders of Hurricane Sandy (*n* = 167; U.S.)	The Disaster Worker Resiliency Training Program (DWRT)	Waitlist RCT testing efficacy while controlling for historical changes (trauma exposure) [*low* risk]	Engagement with healthy lifestyle behaviours; spiritual growth; perceived stress; severity of PTSD symptoms; depression; traumatic exposure
O’Keefe et al., 2024 [32]	American Indian and Alaska Native COVID-19 frontline workers (*n* = 56; U.S.)	Psychosocial skills for COVID-19 Responders, adapted for Frontline Workers in American Indian/Alaska Native Communities	Quasi-experimental (no control) pre-post (1 week and 3 month follow up) observational online pilot evaluation [*high* risk]	Anxiety; burnout; stress; positive mental health; communal mastery; coping skills; PFA knowledge + skills; satisfaction with training; emotional and social well-being
Peng et al., 2024 [33]	Healthcare workers responding to natural disasters (*n* = 1399; China)	System-Based Psychological First Aid Training	A parallel-group, assessor-blinded, cluster RCT (treatment as usual) [*low* risk]	PFA knowledge, skills, + attitude; optimistic self-beliefs; professional quality of life; post-traumatic growth
Reflective Practice				
Travers et al., 2022 [34]	Mental Health and Psychosocial Support Stakeholders (*n* = 37; Europe, Africa, Asia, South America, Australia)	28 (21 with consensus) features of supervision in the context of mental health and psychosocial support	Delphi study pertaining to supervision quality + purpose with bivariate analyses of gender differences [*moderate* risk]	Supervision quality and purpose (not measured)
Mellins et al., 2020 [35]	Healthcare workers (*n* = >1500; U.S.)	CopeColumbia, an intervention intended to bolster emotional well-being and increase professional resilience.	A description of the intervention [*high* risk]	health; health of family + friends; work safety; job responsibilities; financial stability; impact on non-work life (none measured)
Organisational Frameworks				
Weingarten et al., 2020 [36]	Healthcare workers and attorneys (no sample; U.S.)	The Witness to Witness Program for coping with grief, moral injury and distress.	A description the adaption of an intervention to the COVID context [*high* risk]	Witnessing position (qualitatively measured)
Williamson et al., 2018 [37]	NA	Steps to Protect Mental Health in the Face of Moral Dilemmas	Descriptive paper on early support and after-care to reduce moral injury [*high* risk]	Moral injury (not measured)
Umeda et al., 2020 [38]	NA	Recommendations to reduce stressors and stressful situations and improve the management of stress	Literature review and qualitative synthesis of psychosocial supports [*moderate* risk]	Stressors; chronically stressful situations (not measured)
Good Practice Framework, 2020 [12]	NA	Good practice models, principles and recommendations for reducing risk of work-related harm.	A grey literature description of Beyond Blue’s Good Practice Framework [*high* risk]	Work-related harm (not meeasured)
Australian Red Cross, 2020 [39]	NA	Australian Red Cross’ Well-being Support Structure and Methodology	Description of organisational structure, processes to support frontline/emergency workers’ well-being [*high* risk]	Well-being (not measured)
Measures				
Sun et al., 2016 [40]	Firefighters, police, emergency medical technicians and NGOs following a terrorist attack (Germany)	Chinese Compassion-Fatigue-Short Scale (C-CF-SS)	Validation (confirmatory and exploratory) of a translated scale [*low* risk]	Compassion fatigue
Sylwanowicz et al., 2018 [41]	Responders to Typhoon Haiyan (Philippines)	Psychological Simple Triage and Rapid Treatment (PsySTART)	Validation of a scale’s predictive validity for PCL-5 scores four months later (convenience sample) [*moderate* risk]	Depression; PTSD
Rolin et al., 2022 [42]	Healthcare workers (*n* = 423; U.S.)	The Brief COPE Inventory	Validation of a scale’s ability to measure coping style (cross-sectional, convenience sample) [*moderate* risk]	Problem- or emotion-focused coping
Yoon and Choi, 2019 [43]	Mental health professionals, paraprofessionals, disaster site volunteers, related discipline students (*n* = 509; South Korea)	Perceived Competence Scale for Disaster Mental Health Workforce (PCS-DMHW)	Validation (confirmatory and exploratory) of a scale (sampling not fully described, probably convenience) [*moderate* risk]	Individual competence; organisational competence; burnout

Note. CBT = Cognitive Behavioural Therapy; PFA = Psychological First Aid; PTSD = Post-Traumatic Stress Disorder; NGO = Non-Government Organisations; TAU = Treatment as Usual; UK = United Kingdom; RCT = randomised controlled trial.

## Data Availability

Not applicable.

## Abbreviations

The following abbreviations are used in this manuscript:

AI/AN	American Indian and Alaska Native
CISD	Critical incident stress debriefing
CSV	Comma separated values
DWRT	Disaster worker resilience training
PCS-DMHW	Perceived competence scale for disaster mental health workforce
PHQ-8	Patient Health Questionnaire–8-item
PTSD	Post-traumatic stress disorder
REBC	Rational-emotive behavioural coaching
RCT	Randomised controlled trial
